# Perioperative Challenges in Repeat Bladder Exstrophy Repair - Case Report

**DOI:** 10.3889/oamjms.2015.047

**Published:** 2015-07-26

**Authors:** Otu Enenyi Etta, Monday Ituen

**Affiliations:** 1 *Department of Anaesthesia, University of Uyo Teaching Hospital, Uyo, Akwa Ibom State, Nigeria*; 2 *Paediatric Surgery Unit, University of Uyo Teaching Hospital, Uyo, Akwa Ibom State, Nigeria*

**Keywords:** Bladder exstrophy, repair, caudal epidural, analgesia, prolonged surgery

## Abstract

Bladder exstrophy is a rare congenital malformation. It presents as leakage of urine in the anterior abdominal wall following defects in midline anterior abdominal wall skin and bladder. We report the use of combined general anaesthesia and caudal epidural analgesia in a 4yr old boy for repeat bladder exstrophy repair. Problems of prolonged surgery and the challenges of pain and sedation management in the post operative period are discussed.

## Introduction

Bladder exstrophy is a rare congenital malformation of the genitourinary system, with an estimated incidence of approximately 1 per 50,000 live births [1]. The exstrophy-epispadias complex represents a severe midline abdominal birth defect that causes wide separation of the pubic symphysis, an abdominal wall defect and an anteriorly positioned open bladder and urethra [2].

Typically, most bladder exstrophy repairs include closure of the bladder and abdominal wall, and an approximation of the pelvic rami. Postoperatively, children are immobilised in order to promote healing and to maintain pelvic ring integrity. During this time, pain management, nutritional support and meticulous nursing care for osteotomy pin sites, surgical drains and stents, and skin integrity become a focus [3]. A well established interdisciplinary team consisting of surgeons, anesthesiologists, pediatricians, nutritionists, pharmacologists, nurses and child care specialists are essential in providing the environment for successful outcomes [3].

In Nigeria, there is paucity of reports on this rare condition, thus experience in team-approach to its management is lacking. In a recent review in Maiduguri, north-eastern Nigeria, Chinda and colleagues [4] reported a fair outcome in 18 children who underwent bladder exstrophy repairs, however, this report is lacking in perioperative concerns. This was the first case of bladder exstrophy repair in our centre, we report and discuss the perioperative challenges.

## Case Report

A 4 year old male child was scheduled for a repeat single-staged bladder exstrophy repair. Patient had a previous 1^st^ stage surgery at 3^rd^ week of life under general endotracheal anaesthesia. There were no documented anaesthetic complications; however, postoperatively the patient developed leakage of urine at the bladder neck and separation of the pubic bones necessitating a repeat surgery.

**Figure 1:**
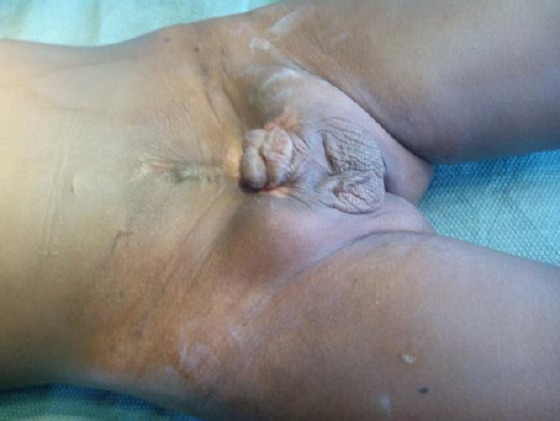
*Bladder exstrophy before surgery*.

The preoperative review revealed no other problems except a lower abdominal scar with suprapubic defect draining urine, pubic diasthesis approximately 5 cm and an epispadias. He weighed 12 Kg and his packed cell volume was 38%, other investigations including intravenous urogram, serum electrolyte, urea and creatinine and renal scan were normal.

**Figure 2:**
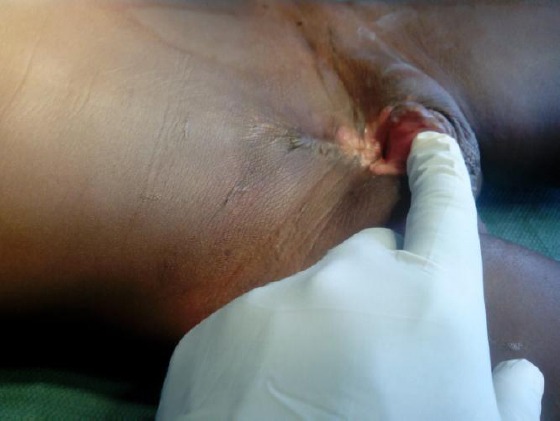
*Bladder exstrophy before surgery*.

Anaesthesia plan consisted of general endotracheal anaesthesia and caudal epidural anaesthesia/analgesia. A multiparameter monitor comprising non-invasive blood pressure (NIBP) and pulse oximeter were attached to the patient and baseline vital signs recorded as follows: pulse rate- 96/m, BP-112/62 mmHg and SP0_2_ -100% on air.

**Figure 3:**
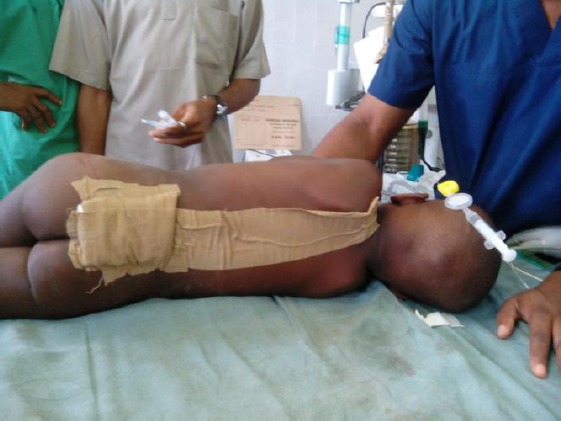
*Caudal epidural catheter taped to the back*.

The patient was preoxygenated for 5minutes and premedicated with 0.24 mg of atropine. Anaesthesia was induced with 50 mg of sodium thiopentone, halothane at 1-2%, and 18 mg of Suxamethonium was given to facilitate endotracheal intubation with size 5mm cuffed endotracheal tube. Caudal epidural anaesthesia was performed using a size 16G tuohy epidural needle. Approximately 4 cm of the catheter was left in the epidural space and loading dose of 8 mls of 1% lidocaine with adrenaline was injected. Halothane was reduced to 0.5%, 6 mg of pentazocine and 0.6 mg of pancuronium were given and surgery was commenced.

**Figure 4:**
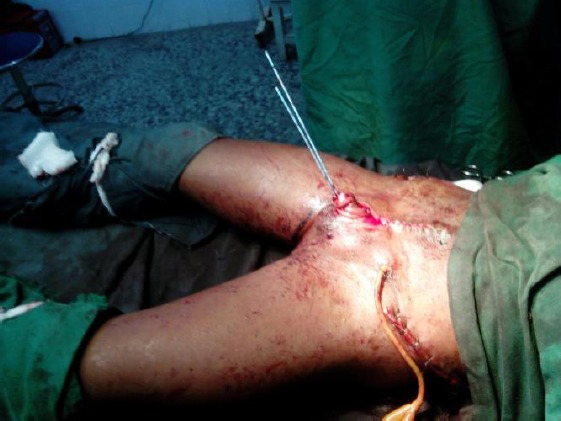
*Immediate postop. showing ureteral stents and bladder catheter*.

Intraoperatively, several procedures were performed by a team of orthopaedic, urologic and paediatric surgeons, these included bilateral anterior innominate bone osteotomy, followed by dissection and delineation of the bladder, insertion of ureteric stents and bladder catheters, repair of the bladder, approximation of pubic bones and epispadias repair.

**Figure 5:**
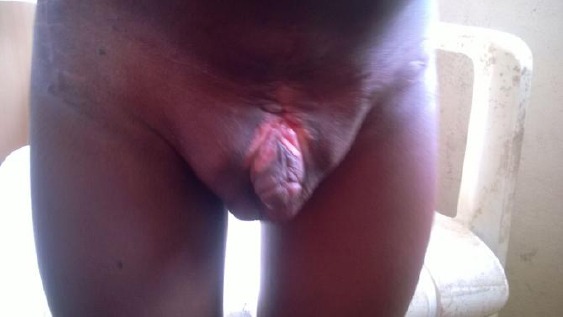
*Bladder exstrophy 3 months post repair*.

Surgery lasted for 10 hrs, the estimated blood loss from gauze count and suction bottle was 250 ml, a total of 250 ml of blood was transfused in 20-40 ml aliquots, approximately 1500 ml of Ringers lactate was used and 1 mg/kg of furosemide was given. At the end of surgery, muscle paralysis was reversed with neostigmine/atropine combination and 150 mg of intravenous paracetamol was given. Patient was extubated in the theatre fully awake and was transferred to the recovery room.

Postoperatively, the patient was managed in the paediatric surgical ward; skin traction was applied to both legs and tied to the foot of the bed. Intravenous fluid and antibiotics were continued, analgesia was maintained with epidural top-up injections using 6ml of 0.125% bupivacaine plus fentanyl 1 µg/ml 6 hrly for 48 hr, rectal diclofenac and intravenous paracetamol. The epidural catheter was removed on the 3rd postoperative day; further analgesia was maintained with oral paracetamol, rectal dicIofenac and intravenous pentazocine as per the surgeon’s order. On the 9th postoperative day, patient developed abdominal wound dehiscence and vesicocutaneous fistula and were managed conservatively to complete healing

## Discussion

The surgical procedure for bladder exstrophy-epispadias complex ranges from modern staged repair of exstrophy (MSRE) to complete primary repair of exstrophy (CPRE) [3-5]. The objectives of treatment have been to achieve a secure closure of the bladder, pelvis and the abdominal wall, preservation of renal function, provision of urinary continence and creation of functional and cosmetically appealing genitalia [5].

Factors that are important for achieving successful primary closure include the use of osteotomy, avoidance of urethral tubes and abdominal distension, the use of postoperative antibiotics, pelvic immobilisation, urethral stenting, catheters and maintenance of the patient free of pain [6, 7]. This was a repeat surgery, failure of the initial surgery may be attributed to the use of Spica cast for immobilisation, poor pain and sedation management as only paracetamol was used for analgesia, as well as inexperience of the nursing staff on the care of exstrophy repair patients. A previous retrospective study showed that use of both spica casting and “mummy wrapping” were associated with lower overall success of the primary closure and higher rate of skin breakdown compared to modified Buck’s traction and Bryan’t traction [7].

Bladder exstrophy repair is a prolonged surgery [8, 9]. Prolong surgery refers to surgery lasting beyond 4-6 hrs [8]. Maintenance of homeostasis during prolonged anaesthesia is a challenge for the anaesthetist. It manifests as disturbances in haemodynamic stability, ventilation, thermoregulation, fluid and electrolyte balance, and acid-base balance etc, therefore, monitoring should be appropriate for the type of surgery. That may include invasive arterial blood pressure monitoring (IABP), central venous pressure (CVP), peripheral capillary oxygen saturation (SP0_2_), end tidal carbondioxide (EtC0_2_), and neuromuscular monitoring. Also, it is important to measure the arterial blood gases (ABG), blood sugar, haematocrit and electrolytes at regular intervals and correct them. If the blood loss is more than anticipated, replacement should be appropriate and coagulation factors should be monitored [8, 9].

In our Patient, the surgery lasted for 10 hr; only pulse oximeter and NIBP were used. Intraoperative monitoring of blood sugar, haematocrits, and other parameters were not done because the facilities were not available, fortunately we did not experience any major anaesthetic complications. Fogarty et al [10], prospectively studied the morbidity associated with prolonged operation time, they found that complications were higher in the patients who underwent head and neck procedures compared to those who had limb surgery. Similarly, Hynynen et al [9], investigated common complications in 22 patients who underwent prolonged plastic surgeries, intraoperative blood loss, hypothermia and breathing difficulties on awakening from anaesthesia were the commonest complications. Our patient developed anemia from blood loss and hypothermia and was treated appropr iately with blood transfusion and warm fluids. Delayed awakening or breathing difficulties were not observed in our patient probably because surgery was done on the lower abdomen and pelvis, and also lower concentrations of halothane was used.

General anaesthesia only was used in the initial repair; we opted for a combined GA and caudal anaesthesia/analgesia because of our intention to participate in the postoperative pain and sedation management, beside the intraoperative benefits of this technique.

The use of caudal epidural analgesia with ropivacaine infusion was reported by Wee and Stoke [11], to offer an excellent postoperative condition in a two day old neonate who underwent bladder exstrophy repair. Similarly, Kost-Byerly et al [12] documented their experience with combined GA and tunneled caudal epidural in 23 newborn infants for bladder exstrophy repair. Postoperatively, a continuous infusion of 0.1% lidocaine was administered for an average of fifteen days, and diazepam for sedation for 20 days, they concluded that perioperative management with tunneled catheter in newborn infants for bladder exstrophy repair facilitates immobilisation, analgesia and sedation, resulting in an excellent cosmetic repair with no case of bladder prolapse or wound dehiscence.

In our patient, the epidural catheter was not tunneled due to our unfamiliarity with the procedure; rather an intermittent bolus injection was used. Epidural catheter was removed on the 3rd postoperative day; leaving caudal epidural catheter beyond 3 days is associated with bacterial colonisation [3]. Our patient developed wound dehiscence and vesicocutaneous fistula which healed without further surgical intervention, these complications may be attributed to the short duration of epidural analgesia and non use of sedatives.

In conclusion, a successful bladder exstrophy repair requires a well coordinated multidisciplinary team approach. Perioperative caudal epidural anaesthesia/analgesia and sedation, meticulous nursing care as well as a team of experienced surgeons improves outcome.
